# Treatment and the Pulmonary Circulation

**Published:** 1895-03-23

**Authors:** Benjamin Ward Richardson


### Treatment and the Pulmonary Circulation.

By Sir Benjamin Ward Richardson, M.D., F.R.S.

### II.—BRONCHIAL CONDENSATION.

I have described the effects of condensation of water in the bronchial passages as occurring in aged people, or in those who are debilitated, and have shown what an important factor such condensation is in the production of dissolution. But it is not alone in these cases that it is injurious. It is often a cause of danger in more acute forms of disease. When the weather is very damp and cold, there is always some risk of condensation of the water of the breath in men and in living and strong animals when they are suddenly checked from strong exercise. We witness in the breath of the horse, when he is checked from a brisk pace, how he pours out a cloud of watery vapour, and it is clear that as he draws in the cold air in inspiration he must condense free quantities of water, derived from his own vesicular lung surface, into his own bronchial passages. Men do the same, and what are called bronchial colds are very frequently "caught," so it is said, in this elementary manner. The mischief increases as the body itself is chilled, and the difficult breathing which we observe under what is called a chill and which is so singularly relieved by removal into a dry, warm air is a complete illustration of the phenomenon to which attention is directed.

There are some acute forms of disease in which *hydrops bronchialis* is developed, particularly in cases where there is obstruction in the upper portion of the bronchial tract, as in laryngitis, croup, diphtheria, and bad quinsy. During conditions in which there is such obstruction, the watery vapour from the vesicular surface of the lung is thrown off with considerable difficulty, and in fact not uncommonly falls back in condensed current from the larger bronchial tubes into the smaller. It is thus that we get, so readily, bronchial complications in the diseases named. The complications consist, first and foremost, of bronchial râles, minute at first in their character, and named by the older observers vesicular râles. Later on there is difficulty of breathing, with cough and expectoration, which is indeed nothing more than a reflexed act for getting rid of fluid producing irritation and obstruction extended and felt through the respiratory centre. If backward condensation still continues, there is exudation of fluid into the parenchymatous tissue with œdema, and, I believe, frequently a form of pleuritic effusion, which is nothing more than a forced attempt under pressure to relieve the lung tissue, through the pleural membrane, of the water with which it is embarrassed. Lastly, in such cases we get the common form of disease known as broncho-pneumonia, which in a large percentage of cases of the kind named is the cause of serious embarrassment, and of death, with the phenomenon vulgarly known as "death rattle." In a case of croup in which death from the laryngeal obstruction took place in two days, I found after death both lungs œdematous, the bronchial tubes charged with aqueous serum, and effusion into the pleural cavities, conditions which seemed to me demonstratively caused by simple condensation. The little patient by the strength of its muscles of inspiration could inspire with fair freedom, but it could not expire with the same facility, inasmuch as the pressure of expiration is so mainly dependent on the elastic tissue, which, in the presence of condensed watery fluid, is reduced in its expulsive power.

Another class of case in which there is bronchial condensation is when the body has been reduced in temperature by the action of certain poisons. All poisons which bring down the temperature of the body rapidly lead to *hydrops bronchialis*, which is the direct and veritable cause of death. The fact is most strongly brought out in a case of poisoning by hydrate of chloral. The most marked effect of this hydrate is to reduce the temperature in all animals that are affected by it. In pigeons, which may have a temperature of 109 deg., I have seen it produce a reduction down to 97 deg., and, in three cases of poisoning by it in the human subject (all of whom, by the way, recovered under warmth) the temperature was reduced to 94 deg. Fahrenheit, and in one to 92 deg. In every patient during this cold stage there was a bronchial râle; in every animal subjected to experiment, where the lungs are structurally the same as in man, there is the state; and in the lungs of animals that have died under the hydrate *hydrops bronchialis* was the striking fact to be detected. They died, in short, drowned in their own bronchial secretion.

One observation more leads to confirmation of the views now stated. In the administration of chloroform by inhalation a tendency to similar condensation of fluid in the bronchial passages is developed, hence the reason why in damp, cold weather the production of the anæsthetic sleep is so prolonged compared with the rapid production observed when the weather is warm, and the air heated and comparatively dry.

The study of the condition of the vesicular structures and of bronchial passages under the various conditions of the air, and under the varied conditions of the body in regard to animal heat and the raising of water into vapour by that heat, becomes a portion of animal engineering which ought ever to be present in the mind of the practitioner. The lungs themselves during life are grand condensing organs; condensers, through the blood, of the oxygen inhaled; condensers, through their own structure, of water inhaled, and at the same time exhalants of powerful degree. Perhaps they are at the same time, by means of their capillary organisation, condensers of blood in the infinite distribution of blood over the vesicles, for it is not improbable that the function of capillary attraction is in health actively in progress in the course of the pulmonic circuit. Be that as it may, we have enough to guide us towards much important observation as to the manner in which, during acute disease, we may do a great deal of good, or a great deal of mischief, in the treatment we adopt, the manner in which we supply air, or the administration of medicines which modify the animal temperature and reduce or increase the propelling power of the heart.

